# A final-year nursing student survey: rural attitudes, perceived competencies and intention to work across five Asian countries

**DOI:** 10.1186/s12912-017-0208-4

**Published:** 2017-03-21

**Authors:** Nareerut Pudpong, Rapeepong Suphanchaimat, Bipin Batra, Jianlin Hou, Lan T. H. Vu, Paul Dipika

**Affiliations:** 1 Healthcare Accreditation Institute (Public Organization), Nonthaburi, Thailand; 2 International Health Policy Program (IHPP), Ministry of Public Health, Nonthaburi, Thailand; 3 National Board of Examinations, New Delhi, India; 4 Institute of Medical Education, Peking University, Beijing, China; 5 Department of Epidemiology, Hanoi school of Public Health, Hanoi, Vietnam; 6 Research, Training and Management (RTM) International, Dhaka, Bangladesh

**Keywords:** Attitude, Competencies, Intention to work, Rural areas, Nursing students, Public service

## Abstract

**Background:**

Shortages and maldistribution of nurses remain significant problems in many countries. Having appropriate intervention strategies to retain nurses in underserved areas, where they are most needed, are crucial for health system strengthening. This study aimed to quantify attitudes to working in rural areas, perceived competencies, and intention to work among final-year nursing students, and to analyze the associations between those factors and their background characteristics across five countries in the Asia-Pacific Network for Health Professional Education Reforms (ANHER), namely Bangladesh, China, India, Thailand, and Vietnam.

**Methods:**

A descriptive comparative cross-sectional survey was conducted between July 2012 and July 2013, using a self-administered questionnaire to assess students’ attitudes towards working in rural areas, their perceived competencies, and their intended job choices. A total of 10,169 final-year nursing students in five countries were selected. Bivariate models were constructed to compare students’ characteristics. Statistically significant variables were further analyzed using multivariate models.

**Results:**

Most nursing students in five countries had rural backgrounds. Students in India (67.1%) and Thailand (65.1%) held more positive attitudes towards working in rural areas. Students in Bangladesh (78.8%) and India (62.6%) believed that their schools prepared them well, and inspired them, to work in rural areas. The ‘Lifelong learning’ competency was ranked highest by students in all five countries, ranging from 76.2 to 91.7%. Their perceived competencies were significantly related to their background of having graduated from rural high schools and being admitted to study through rural recruitment. Rural upbringing and rural recruitment were significantly associated with more positive attitudes towards rural areas (*p*-value < 0.5). A majority of students in China (83.8%), Thailand (67.7%) and Vietnam (86.5%) intended to work in the public sector immediately after graduation.

**Conclusions:**

These findings from five Asian countries confirm that nursing students with rural upbringing and recruitment had more positive attitudes toward rural areas and were more likely to choose working in rural areas after graduation. This study provides additional evidence from country implementation to support the value of WHO recommendations of effective strategies to address issues of rural retention by focusing on the recruitment of students with a rural background.

## Background

Nurses constitute the majority of the health workforce in the health care system of most countries in the Asia. An adequate, competent and motivated nursing workforce can generate effective health outcomes [1]. A shortage of nurses was one of the major nursing workforce issues in the region, which has been further aggravated by migration [2]. Nurse training institutions can play a vital role in developing a national nursing workforce plan and in producing an adequate number of competent nurses for health systems. Therefore, understanding nurses’ attitudes, competencies and intention to work during training and/or after graduation is useful for developing appropriate intervention strategies to retain nurses in health care systems, particularly in underserved areas. This information will also be helpful for educational institutions seeking to develop appropriate and relevant curricula to promote positive attitudes towards working in rural/underserved areas, to encourage the intention to work in the public sector, and to enhance the competencies of nursing students to ensure that they can respond to the health needs of the populations they serve.

Previous literature has suggested that medical and nursing students born or brought up in rural areas are more likely to choose to work in rural areas [3–5]. This may be because their positive attitudes toward rural practice and environments have gradually developed since childhood and remained significant factors influencing their job choices after graduation. Studies conducted among newly graduated doctors in Thailand and other lower-middle income countries (LMICs) showed that students admitted to medical schools through a special quota for students with a rural-background were more likely to choose working in community hospitals in rural areas [4–6]. A recent study conducted among Thai nursing students also revealed that students’ attitudes towards working in rural areas is a strong predictor of their intention to work in rural areas [7]. The World Health Organization (WHO) has also recommended that rural recruitment is an effective human resources for health (HRH) strategy to address rural retention [8].

In addition, it is also important to attract and retain health workers in the places where they are most needed, to improve quality and safety in health care systems. Thus, there is a need to increase competencies among health professionals. Patient-centered care, teamwork and collaboration, evidence-based practice, quality improvement, safety, and informatics are examples of necessary competencies required for health system improvement [9]. It has been pointed out that poor productivity in health care systems may be due to the lack of adequate competencies within the health workforce [10]. The competencies of health care workers can be enhanced prior to the delivery of services through the provision of appropriate training activities to build up essential skills before graduation. The assessment of students’ self-perceived competencies is one way to gather information that can help in the improvement and/or adjustment of curricula to ensure their suitability for students’ needs.

In 2013, WHO and WHO-Southeast Asia Regional Office (WHO-SEARO) adopted resolutions to move the agenda for transforming health workforce education in support of universal health coverage [11]. Consequently, the Asia-Pacific Network for Health Professional Education Reforms (ANHER) was formed to develop a standard protocol and tools for assessing health professional education. ANHER, a five-country network, including Bangladesh, China, India, Thailand and Vietnam, developed and implemented suitable standard tools for undertaking health workforce education assessments in health professional education, including the nursing education assessment presented in this paper.

In Bangladesh, there are two forms of pre-service education in nursing; the first is the Diploma in Nursing. There are 43 public nursing institutes providing a total of 2,580 training places, and 107 private nursing institutes delivering training to a maximum number of 5,415 students per year. The second form is the BSc in Nursing. This degree is available in 18 public nursing colleges offering a total of 1,435 training places, and 31 private nursing colleges that can produce up to 2,605 graduates per year. The majority of nursing training institutions are located in urban areas. In addition, there is an in-service (post-basic) training, which seeks to build the capacity of nurses to become nursing managers, nursing teachers, nursing administrators, and nursing leaders; this is available in four colleges, offering a maximum of 500 training places. Specialized short-course training has also been provided, such as Coronary Care Unit (CCU)/Intensive Care Unit (ICU) and Cardiac Nursing in the National Heart Foundation and Rehabilitation Nursing in the Bangladesh Health Professions Institute (BHPI), Centre for the Rehabilitation of the Paralysed (CRP), Savar. However, each course is limited to only 20 nurses per year. The nursing profession in Bangladesh has expanded with the active involvement of both public and private sector organizations and is gaining momentum to cope with the increasing demand from, and needs of, the health sector. At present, there are approximately 20,000 registered nurses in Bangladesh of whom 14,686 nurses work for the Government [12]. The remainder work in either the domestic private sector or work abroad in countries such as the UK, USA, or in the Middle-East countries. The nursing profession in Bangladesh is facing challenges regarding their lower position as compared to other professions. Traditionally, nurses were viewed as support service providers and, thus, undervalued. However, the nursing profession has been upgraded as a second class job (at the start) and the nurse’s role was redefined as a member of the service delivery team in 2011 [13]. Nevertheless, the view of being a lower prestigious profession still remains and makes it difficult to recruit more nurses. Other barriers to recruiting more nurses include a failure to attract wealthy and/or higher-status people to join the nursing profession, misconceptions regarding nursing, socio-cultural attitudes, relatively lower salaries, and domination by the other professionals in the health care team.

In China, nurses are the second largest group of health professionals in the country. The nursing education system in China is complex in terms of institutional characteristics, such as affiliation, ownership, location, and management models. Essentially, there are two kinds of nurse producers, namely, secondary health schools, and colleges/universities. The former belongs to middle education and their graduates are offered a diploma, called a *Zhongzhuan* degree. The latter, which may be free-standing colleges or comprehensive universities, is a part of higher education. Depending on the length of their training, their graduates may be offered a *Dazhuanor* junior college (diploma), bachelor’s, master’s, or doctoral degree. In 2007, enrollment in nursing training totalled 391,000 students, of whom71% were *Zhongzhuan* students and the remainder were students who studied in *Dazhuanor,* or higher, programs [14]. Most of nursing schools in China are located in urban areas. As in other countries, the shortage of nurses has been a long-standing issue that continues to affect the performance of the health system in China. This problem was exacerbated by the fact that most nurses in China prefer to work in urban areas, leading to a serious shortage of nurses in low-resource areas. Furthermore, nurse retention is of great concern in both urban and rural areas. Compared with physicians, nurses are more likely to quit their job or migrate, for various reasons, including better salaries, better working conditions, or better career opportunities.

In India, there are 1,690 colleges offering a BSc in nursing. Of these colleges, 117 are public institutions and 1,573 are private. These colleges offer 84,942 places per year for nursing students [15]. Nursing education in India is not streamlined and therefore faces several challenges. A streamlined course consisting of a unified and comprehensive syllabus replacing different levels of courses, such as a diploma course, including Auxiliary Nurse Midwife (ANM) and General Nursing and Midwifery (GNM), and a BSc in nursing, should be introduced. A few states offer diploma-level courses in addition to the basic level course of BSc in nursing [16]. Nursing schools in India are prevalent in both urban and rural areas of the country. However, many smaller states of the northeast and other less-developed regions lack the basic infrastructure required to provide a complete and updated nursing education. For nursing in primary health centres and district hospitals, the focus is on maternal and child health care and optimal nutrition. With regard to epidemiological changes, the need for specialties like non-communicable diseases, and environmental and occupational health hazards, has also emerged. To tackle these emerging problems effectively, it is necessary to develop specialized skills among health professionals [16]. In addition, HRH challenges in India are also related to maldistribution. Nationally, there are only 0.9 nurses per 1,000 population, with higher numbers of nurses and midwives in urban areas (1.59/1000 population) than rural areas (0.41/1000 population) [16].

In Thailand, there are 85 nursing schools (public 62, private 23) which produce approximately 10,000–12,000 nurses annually [17, 18]. The Thai health system is dominated by the public sector, with 75% of registered nurses (RNs) working for the Ministry of Public Health (MOPH), and serving both urban and rural areas [18]. Due to rapid economic growth and competitiveness between sectors in the health care market, there has been an expansion of the private healthcare sector. The “pull factors” for nurses are high in the private sector, for example, better amenities, higher salaries, greater possibilities of accessing higher education and more opportunities for dual practice. Over time, these pull factors could lead to an imbalance of nurses between the public and private sectors with an increasing number of nurses choosing to work in the private sector. Thus, the critical concern among policy makers is not only an intervention to attract nurses to practice in rural areas, but also to retain them in the public sector. Thailand has employed two major strategies to maintain a sufficient number of nurses working in the public sector: firstly, by geographically distributing nursing institutions for training student nurses in rural areas as most schools are located outside the capital city, Bangkok (but still in the central area of a province) and, secondly, by introducing a rural recruitment policy for public nursing institutions to promote increased admission of students with rural backgrounds. Since the demand for nurses in the urban private sector is still increasing, there is concern that, in the long term, these two strategies alone may not be sufficient to retain nurses in the public health system.

In Vietnam, there are a total of 123 nursing training schools and, of these, 18 provide bachelor’s degrees and the rest offer college/secondary training programs (which provide a course certificate, not a degree). Most of these nursing schools are in the public sector and are located in urban areas. The private nursing training system remains undeveloped (only eight private universities have nursing faculty providing bachelor’s degrees and ten schools/departments provide nursing training at the secondary level). Vietnam has a total of 83,369 nurses with about 5.7% working in the private sector [19]. On average, Vietnam has10.7 nurses per 10, 000 population (in 2013). As is the case with other cadres of health worker, there is an uneven distribution of the nursing workforce between rural and urban areas. The urban population is only 28.4% of the total population, but 57% of nurses in Vietnam work in urban areas. The quality of nurses is also an issue because the proportion of nurses with a university degree was only 0.1–0.3 per 1,000 population. Due to changes in the burden of disease and demography, the need for high quality nursing services is increasing significantly. The population is in need of nursing home services, so the numbers of nursing home and public health nurses are expected to rise dramatically. To meet the demand for health care services, it is necessary to develop nursing programs specialized in home care or community-based care in Vietnam.

Overall, a shortage of skilled nurses and midwives remains an issue in Bangladesh, China and Vietnam, and the maldistribution of nurses between urban and rural areas is an important issue of concern in all five studied countries. Understanding attitudes, competencies, and the intended work location among nursing students may help inform policy as well as helping in the design of interventions that would encourage nurses to choose to work in rural areas where they are most needed.

This paper therefore aims to describe the attitudes, competencies and intention to work among final-year nursing students and identify factors associated with those characteristics across the five selected countries in Asia.

## Methods

### Population and survey tool

A cross-sectional survey was performed between 1 July 2012 and 1 July 2013 using a structured, closed ended questions, self-administered questionnaire. Participants of the survey were final-year nursing students in the five countries. The total number of participants was 10,169, which is broken down as follows: 898 from Bangladesh (BGD), 2,218 from China (CHN), 2,880 from India (IND), 3,349 from Thailand (THA), and 820 from Vietnam (VNM). These figures correspond to response rates of 98.9% (BGD), 90% (CHN), 99% (IND), 67.6% (THA) and 89.3% (VNM) respectively.

The questionnaire was developed mainly in Thailand; however researchers from all five countries were involved from the early stage of questionnaire development. A pilot test was conducted among 30 nursing students in Thailand. The pilot study yielded Cronbach’s alpha coefficient of 0.79, suggesting acceptable reliability. A series of consultative meetings between principal investigators from all countries was arranged. This process was done to ensure content validity and allow researchers in each country to adapt the wording/text in the questionnaire to meet their local context while keeping the core idea/intention.

The questionnaire is composed of four sections: (1) the personal attributes of respondents, (2) attitudes to working in rural areas, (3) clinical and public health competencies, and 4) job preference after graduation. Section 1 asked respondents to specify their age, sex, the location of the town in which they spent their childhood (rural vs semi-urban vs urban), the location of their parents’ residence (rural vs semi-urban vs urban), the location of their high school (rural vs semi-urban vs urban), their mode of admission to nursing education (national entrance examination vs non-national entrance e.g. special quota, direct admission), their parents’ level of education (below bachelor degree vs at least bachelor degree) and their parents’ occupation (professionals vs non-professionals). It should be noted that the definition of ‘urban’, ‘semi-urban’ and ‘rural’ varied according to the context of each country.

In Section 2, participants were asked to specify (2.1) their attitudes towards working in rural areas, and (2.2) their attitudes towards their nursing school, specifically whether or not they prepared students well for performing clinical work in rural areas. The answers to these questions were formulated as binary choices, namely, ‘positive attitude’ or‘not-positive attitude’.

Section 3 was made up of a list of five competencies: (5.1) critical thinking and evidence-based practice, (5.2) professional competencies, (5.3) leadership skills, (5.4) management and collaboration skills and (5.5) lifelong learning. Like Sections 2 and 3, the answers were restricted to binary choices, namely, ‘confident’ and ‘not confident’ in each competency.

The final section consisted of two sub-questions: (3.1) job preference immediately after graduation, and (3.2) job preferences in 5 years’ time. The answers to these questions were also binary: namely, ‘pro-public’ and ‘not pro-public’.

The language used for the survey was the communication language used in each country, which was Chinese for China, English for Bangladesh and India, Thai for Thailand, and Vietnamese for Vietnam. Each country co-author took responsibility for checking the translations (back and forth several times) to ensure quality before data collection. Each co-author also had their own focal point, who was the officer at the target nursing colleges/universities, and was well-trained in the study protocol. This officer was responsible for approaching nursing students at their classrooms, ask if they were willing to participate with informed consent, and then collecting the completed questionnaires and returning them to the country co-author.

### Data analysis

STATA software Version XI was used for data analysis. The analysis consists of three parts. Firstly, descriptive statistics were used to describe the demographic profiles of graduates and the general findings regarding attitudes to working in rural areas, workplace preferences and competencies by country. Secondly, bivariate analysis was performed, using Pearson’s Chi-square test, to determine an association between each independent variable (individual attributes) and each outcome variable (attitudes to working in rural areas, workplace preference and competencies). Finally, multivariate analysis was conducted to explore the effect size of the outcome variables while adjusting all potential confounders simultaneously. The independent variables selected in the model were those yielding statistical significance above a 95% level of confidence in the bivariate analysis. The results are presented in terms of odds ratios. The statistical model applied in this section was a multilevel mixed-effect logistic regression model, which was the random intercept model. Results were adjusted by ‘country’, which was considered as a higher hierarchical variable.

## Results

The sample size (n) of this comparative study was 10,165 nursing students drawn from the five countries. The majority of participants were female aged 22–23 years. Over 80% of respondents across these countries had been admitted to study through measures other than the standard national entrance examination, e.g., special quota and direct admission. Nursing students who had spent their early childhood in rural areas made up between 39–81% of the respondents. For their high school study, these students tended to move to schools in more urban areas. For example, in Vietnam, 81% of students had resided in a rural area, but this number decreased to only 47% who had attended high school in a rural area. The demographics of students’ parents were quite similar across the five countries. A significant proportion of parents lived in rural areas, ranging from 39–79%. The majority of parents were classified as non-professionals (67–90%) with a diploma or lower degrees of education (69–96%), see Table 1.

**Table 1:** Characteristics and background information of participants

		Bangladesh	China	India	Thailand	Vietnam
Sample size (n)		898	2,218	2,880	3,349	820
Mean age in years (s.d.)		22.1 (3.8)	23.8 (1.2)	23.3 (2.9)	23.0 (1.2)	23.1 (0.8)
Sex (%)	Female (%)	93.3	91.5	92.4	94.7	87.1
Mode of admission (%)	Other modes	95.6	87.0	82.1	84.5	85.2
	National entrance	4.4	13.0	17.9	15.5	14.8
Domicile during childhood period (%)	Rural	74.1	52.8	39.4	49.6	81.1
	Semi-urban	11.5	26.3	27.7	29.8	10.6
	Urban	14.4	20.9	32.9	20.6	8.3
Domicile of high schools (%)	Rural	68.2	18.1	25.2	11.9	46.9
	Semi-urban	17.4	54.7	36.5	32.9	41.9
	Urban	14.4	27.2	38.2	55.2	11.2
Domicile of parents (%)	Rural	75.6	51.2	39.0	49.2	79.0
	Semi-urban	9.2	27.5	25.1	31.5	10.7
	Urban	15.2	21.3	35.9	19.3	10.3
Father’s occupation (%)	Non-professionals	66.9	86.0	78.0	79.1	84.4
	Professionals	33.1	14.0	22.0	20.9	15.6
Mother’s occupation (%)	Non-professionals	89.8	87.3	88.1	85.2	89.6
	Professionals	10.2	12.7	11.9	14.8	10.4
Father’s education (%)	Diploma or below	81.6	81.4	68.6	80.9	78.5
	Bachelor or above	18.4	18.6	31.4	19.1	21.5
Mother’s education (%)	Diploma or below	95.6	87.0	82.4	84.5	85.2
	Bachelor or above	4.4	13.0	17.6	15.5	14.8

### Attitudes towards working in rural areas, self-perceived competencies, and job preferences

Students were asked about their attitudes towards working in rural areas and the role of their school in preparing them for work in rural areas. The percentage of students with a ‘positive attitude’ towards working in rural areas was above 60% in India and Thailand, whereas the attitude towards their school’s role in preparing them to work in rural areas were relatively high in Bangladesh and India at 78 and 62% respectively.

Participants also rated their level of confidence in five main competencies which are required when working in rural areas, namely: critical thinking and evidence-based practice, professional competencies, leadership skills, management and collaboration skills, and lifelong learning. More than half of the surveyed nursing students across the five countries showed the same level of confidence in two competencies: management and lifelong-learning. Students of all countries, except for Vietnam, had confidence in the leadership competency (59–73%). Except for those in India, most students had confidence in their critical thinking capability and had a pro-public job preference once they graduate and, for the point in time 5 years after, was even higher.

The preferences for work immediately after graduation for nursing students from China and Vietnam were identified as ‘pro-public’. The percentage of students who anticipated holding a ‘pro-public’ job preference 5 years after graduating was lower than immediately after graduation in four of the studied countries; the exception was Bangladesh where the percentage of students with a ‘pro-public’ preference increased from 56% immediately after graduation to 66% after 5 years, Table 2.

**Table 2:** Rural attitude, perceived confidences, and intention to public work of participants, percent

		Bangladesh	China	India	Thailand	Vietnam
1. Positive attitude towards …						
Rural working	No	46.3	60.7	32.9	35.0	50.2
	Yes	53.7	39.3	67.1	65.1	49.8
Role of School in preparing to work in rural areas	No	21.2	69.0	37.4	52.2	68.8
	Yes	78.8	31.0	62.6	47.8	31.2
2. Perceived confidence in …						
Critical thinking competency	No	15.8	48.0	50.2	34.5	43.9
	Yes	84.2	52.0	49.8	65.5	56.1
Professional competency	No	35.3	48.8	51.4	42.4	55.0
	Yes	64.7	51.2	48.6	57.6	45.0
Leadership competency	No	40.3	41.3	41.9	26.5	57.1
	Yes	59.7	58.7	58.1	73.4	42.9
Management competency	No	21.2	35.7	39.4	20.8	42.3
	Yes	78.8	64.3	60.6	79.2	57.7
Lifelong-learning competency	No	11.7	23.8	21.9	8.3	20.2
	Yes	88.3	76.2	78.1	91.7	79.8
3. Intention to ‘pro-public’ work (job preference)						
Immediate	No	44.0	16.2	58.5	32.3	13.5
	Yes	56.0	83.8	41.5	67.7	86.5
For next 5 years	No	33.7	36.5	61.5	38.3	17.6
	Yes	66.3	63.5	38.5	61.7	82.4

Response variables which were statistically significant in bivariate models were selected to be analyzed using multivariate analysis. Table 3 summarizes the results of these nine dependent variables, which consisted of “attitude towards working in rural areas” (2), confidence in their competencies (5), and extent of pro-public sector job preference (2). Growing up in an urban area clearly influenced students’ attitudes towards both working in rural areas and towards the role of schools in preparing students for that rural work.

**Table 3:** Percentage and *p*-value of rural attitude, perceived competencies, and intention to public work against background characteristics

	Positive attitudes towards				Perceived competency in:										Job preference pro-public sector			
	Working in rural areas		Role of school in preparation for rural work		Critical thinking		Professionalism		Leadership		Management		Lifelong-learning		Immediately		5 years’ time	
	No	Yes	No	Yes	No	Yes	No	Yes	No	Yes	No	Yes	No	Yes	No	Yes	No	Yes
Sex	*P* = 0.172		*P* = 0.956		*P* = 0.124		*P* = 0.046		*P* = 0.119		*P* = 0.035		*P* = 0.657		*P* = 0.022		*P* = 0.038	
• Male	7.8	7.1	7.4	7.4	6.9	7.7	6.9	7.9	6.9	7.7	8.2	7.1	7.7	7.4	8.2	6.9	8.0	6.9
• Female	92.2	92.9	92.6	92.6	93.1	92.3	93.1	92.1	93.1	92.3	91.8	92.9	92.3	92.6	91.8	93.1	92.0	93.1
Age in years	*P* = 0.002		*P* < 0.001		*P* = 0.733		*P* = 0.014		*P* = 0.003		*P* = 0.946		*P* = 0.258		*P* < 0.001		*P* = 0.4164	
Domicile during childhood period	*P* < 0.001		*P* = 0.005		*P* < 0.001		*P* = 0.002		*P* = 0.015		*P* = 0.431		*P* = 0.217		*P* < 0.001		*P* < 0.001	
• Rural	55.2	49.8	51.6	52.6	49.8	53.7	52.1	52.1	53.8	51.1	51.5	52.4	50.2	52.5	45.7	55.7	45.9	56.6
• Semi-urban	24.6	25.8	26.6	23.9	26.3	24.6	26.5	24.1	24.9	25.5	26.1	24.9	26.4	25.0	26.5	24.5	27.2	23.9
• Urban	20.2	24.4	21.8	23.5	23.9	21.8	21.4	23.8	21.3	23.4	22.4	22.7	23.4	22.5	27.8	19.8	26.9	19.5
Domicile of high schools	*P* < 0.001		*P* < 0.001		*P* = 0.004		*P* < 0.001		*P* < 0.001		*P* < 0.001		*P* < 0.001		*P* < 0.001		*P* < 0.001	
• Rural	26.3	23.8	21.6	28.2	23.6	25.7	25.3	24.5	28.0	22.9	26.0	24.4	26.4	24.6	23.4	25.6	21.3	27.4
• Semi-urban	40.1	36.5	39.9	36.1	39.8	36.8	39.6	39.6	38.9	37.5	40.5	36.9	40.9	37.4	35.0	39.9	37.3	38.6
• Urban	33.6	39.7	38.5	35.7	36.5	37.5	35.1	38.9	33.1	39.6	33.5	38.7	32.7	38.0	41.6	34.5	41.4	34.0
Domicile of parents	*P* < 0.001		*P* = 0.038		*P* < 0.001		*P* = 0.025		*P* = 0.036		*P* = 0.249		*P* = 0.121		*P* < 0.001		*P* < 0.001	
• Rural	53.9	49.7	50.6	52.4	48.8	53.3	50.9	51.9	53.1	50.5	50.3	52.0	49.5	51.9	44.9	55.1	44.9	56.1
• Semi-urban	25.0	25.3	26.2	24.1	6.2	24.4	26.4	24.1	24.3	25.6	26.1	24.8	25.5	25.1	26.2	24.6	26.9	24.0
• Urban	21.1	25.0	23.2	23.5	25.0	22.3	22.7	24.0	22.5	23.9	23.6	23.2	25.0	23.0	28.9	20.3	28.2	19.9
Father’s occupation	*P* = 0.040		*P* = 0.027		*P* = 0.656		*P* = 0.452		*P* = 0.923		*P* = 0.892		*P* = 0.623		*P* < 0.001		*P* = 0.001	
• Non-professionals	80.6	78.9	80.6	78.8	79.4	79.8	80.0	79.4	79.6	79.7	79.7	79.6	79.2	79.8	77.5	81.0	78.2	80.9
• Professionals	19.4	21.1	19.4	21.2	80.6	20.2	20.0	20.6	20.4	20.3	20.3	20.4	20.8	20.2	22.5	19.0	21.8	19.2
Mother’s occupation	*P* = 0.221		*P* = 0.016		*P* = 0.012		*P* = 0.551		*P* = 0.750		*P* = 0.168		*P* = 0.589		*P* = 0.051		*P* < 0.001	
• Non-professionals	87.8	86.9	86.5	88.1	86.3	88.0	87.1	87.5	87.1	87.4	86.6	87.6	86.9	87.4	86.4	87.8	85.6	88.5
• Professionals	12.2	13.1	13.5	11.9	13.7	12.0	12.9	12.5	12.9	12.6	13.4	12.4	13.1	12.6	13.6	12.2	14.4	11.5
Father’s education	*P* < 0.001		*P* = 0.100		*P* = 0.025		*P* = 0.711		*P* = 0.720		*P* = 0.023		*P* = 0.184		*P* < 0.001		*P* = 0.001	
• Diploma or below	79.4	75.8	78.0	76.7	76.2	78.1	77.5	77.2	77.2	77.5	75.9	78.0	76.1	77.6	75.2	78.6	75.7	78.6
• Bachelor or above	20.6	24.2	22.0	23.3	23.8	21.9	22.5	22.8	22.8	22.5	24.1	22.0	23.9	22.4	24.8	21.4	24.3	21.4
Mother’s education	*P* = 0.086		*P* = 0.039		*P* = 0.006		*P* = 0.444		*P* = 0.765		*P* = 0.010		*P* = 0.022		*P* < 0.001		*P* < 0.001	
• Diploma or below	86.2	85.0	84.8	86.2	84.3	87.3	85.8	85.2	85.6	85.4	84.1	86.1	83.7	85.8	83.6	86.6	83.4	87.1
• Bachelor or above	13.8	15.0	15.2	13.8	15.7	13.7	14.2	14.8	14.4	14.6	15.9	13.9	16.3	14.2	16.4	13.4	16.6	12.9
Mode of admission	*P* < 0.001		*P* < 0.001		*P* = 0.045		*P* = 0.381		*P* < 0.001		*P* = 0.001		*P* = 0.002		*P* < 0.001		*P* < 0.001	
• Other modes	39.9	53.8	39.7	56.3	46.7	48.8	47.5	48.3	44.7	49.9	45.4	49.0	44.6	48.6	64.6	38.5	56.4	41.4
• National entrance	60.1	46.2	60.3	43.7	53.3	51.2	52.5	51.7	55.3	50.1	54.6	51.0	55.4	51.4	35.4	61.5	43.6	58.6

Enrolling in a school in an urban or semi-urban area made students less likely to intend to work in public facilities after graduation. Having parents residing in urban areas also had a similar effect of corresponding with a higher preference from students towards working in private institutions. Having been admitted to study through a national entrance exam was also a strong predictor of intention to work in public, rather than private, facilities (Table 4).

**Table 4:** Multivariate analysis for positive attitude towards working in rural and pro-public preference

	Positive attitudes towards working in rural (model 1)		Immediate job preference with pro- public (model 2)		Preference for the next 5 years with pro-public (model 3)	
	Odds Ratio	[95% CI]	Odds Ratio	[95% CI]	Odds Ratio	[95% CI]
Female (VS male)			**1.374	[1.152–1.638]	*1.227	[1.040–1.448]
Childhood domicile (VS rural)						
• semi-urban	1.099	[0.927–1.302]	1.089	[0.905–1.310]	1.055	[0.889–1.252]
• urban	*1.272	[1.053–1.538]	0.969	[0.795–1.182]	1.041	[0.862–1.257]
High school domicile (VS rural)						
• semi-urban	1.029	[0.909–1.164]	0.890	[0.774–1.024]	*0.867	[0.762–0.987]
• urban	0.983	[0.848–1.140]	**0.789	[0.672–0.926]	**0.766	[0.659–0.890]
Parent domicile (VS rural)						
• semi-urban	0.913	[0.772–1.080]	**0.782	[0.652–0.938]	*0.829	[0.701–0.982]
• urban	0.956	[0.800–1.147]	0.837	[0.693–1.012]	*0.824	[0.688–0.986]
parent being profession (VS not profession)	1.020	[0.912–1.141]	0.967	[0.859–1.088]	*0.874	[0.780–0.978]
parent having bachelor degree or above (VS having diploma or below)	1.053	[0.942–1.178]	1.024	[0.910–1.153]	1.029	[0.919–1.152]

*p < 0.05, **p < 0.01

• Random effects parameter (95% CI) for model 1 = 0.416 (0.222, 0.780)

• Random effects parameter (95% CI) for model 2 = 0.828 (0.443, 1.546)

• Random effects parameter (95% CI) for model 3 = 0.596 (0.318, 1.117)

## Discussion

### Attitudes towards working in rural areas

Overall, the final-year nursing students surveyed across the five studied countries displayed relatively positive attitudes towards working in rural areas, ranging from approximately 50–70%, with only one exception, China, which had the lowest percentage of 39%. In terms of inspiration to work in rural areas, nursing students in Bangladesh and India gave higher percentages, above 50%, indicating that they felt better-prepared to work in rural areas than students from the other three countries, all of which were lower than 50%.

This study found that nursing students in India (67.1%) and Thailand (65.1%) held more positive attitudes towards working in rural areas than other three studied countries. It is interesting that, while Indian nursing students in this study had positive attitudes toward working in rural areas, another survey conducted in 2010 found that Indian nursing students considered that supportive environments, particularly, basic facilities like 24-h water and electricity, good sanitation, and clean surroundings were scarce in rural areas [20]. This suggests that such deprivations in rural areas may not prevent Indian students from choosing to work in rural areas if they learn about and understand the situation in those areas. This study also found that nursing students in Bangladesh (78.8%) and India (62.6%) agreed that their schools prepared them well, and inspired them, to work in rural areas. Since most training institutions in these five countries are located in urban areas (except only some nursing schools outside the capital city of India and Thailand), introducing more time and exposure to rural experiences and environments during training would be crucial to help provide knowledge and understanding about rural practices and encourage students to choose working in rural areas after graduation. This corresponds to the theory-based model of Ajzen and Fishbein that has indicated that the intention to work was generally influenced by attitudes and subjective norms and so could be impacted during their training [21].

When looking at factors associated with attitudes to working in rural locations, it was found that a rural upbringing was also significantly associated with more positive attitudes towards rural practice, which were about 1.2 times higher than those of students with an urban upbringing (*p*-value < 0.5). This finding is consistent with studies conducted in Australia, the Philippines, and Canada, which found that students who were born in rural areas, and trained in schools located in rural settings, were more likely to choose to work in rural areas [22, 23]. It is possible that this more positive attitude comes from students’ familiarity with rural life and rural environments. Having a father with a higher level of education was significantly associated with students having more positive attitudes toward rural practice (OR = 1.13) compared to those whose fathers had a lower level of education (*p*-value <0.05). In general, fathers are role models for their children, however, it is unclear why a father with higher education could lead to more positive attitudes towards rural practice among nursing students. This finding contradicts a study conducted in Korea, which found that new nurse graduates who worked in non-metropolitan hospitals were those whose fathers had less than 4 years college education [24]. Understanding this more clearly will require future studies. In addition, some other factors, such as fathers’ involvement in their children’s development or a student’s core values, should be included in future analysis.

### Perceived competency

When looking at the key perceived competencies required to work in rural areas, nursing students in all five countries were shown to have more confidence in the “Lifelong learning” competency, but needed relatively more support to attain confidence in the “Professional and Leadership” competencies.

The “Lifelong learning” competency was ranked highest by students in all five countries, ranging from 76.2 to 91.7%. Students in Bangladesh had higher “critical thinking” (84.2%) and “professional” competencies (64.7%), while Thai students had higher leadership (73.4%) and management skills (79.2%). It was also found that their perceived competencies were significantly related to their background having graduated from rural high schools or having been selected through a rural recruitment program. This finding is relatively similar to the finding among dental graduates in Thailand that found that students who graduated from schools located outside Bangkok and the vicinity were more confident with their competency in public health than those who graduated from schools within metropolitan Bangkok [25]. Results of a study conducted among final-year nursing students in Australia also suggest that encouraging students to undertake rural exposure experiences helped develop their confidence and competencies, and this was likely to increase their willingness to work in rural areas [26]. A likely explanation for this phenomenon is that students that spend their clinical years in a rural area are likely to have hands-on experience and exercise their management skills more than those in an urban area. This is consistent with a study by Putthasri et al.[6], which reported that patients presenting at university medical school hospitals (mostly in urban areas) tended to have more complicated conditions, which required specialist care. Such a situation limits chances for undergraduate students to handle or manage care for patients independently [6]. Thus, providing greater exposure to rural practice and rural environments may not only enhance students’ confidence and levels of competency, but it may also encourage them to select a rural placement.

### The intention to work in the public sector

Higher numbers of students in China (83.8%), Thailand (67.7%) and Vietnam (86.5%) intended to work for the public sector immediately after graduation, while approximately half of nursing students in Bangladesh (56.0%) and India (41.5%) intended to work in the public sector. For Bangladesh, this may be explained by the lower level of prestige associated with the nursing profession in comparison to other professions, as well as a lack of government positions for nurses. Similarly, in India, nurses are more likely to work for the government only if there is a permanent position available for them.

The percentage of students with anticipated ‘pro-public’ job preferences 5 years post-graduation tended to decrease in four of the five countries -- the exception was Bangladesh where the percentage of ‘pro-public’ preferences increased from 56%, immediately after graduation, to 66% when anticipating 5 years in the future. This may be because public jobs in Bangladesh are considered to provide high levels of job safety and security when compared to private sector jobs. In addition, since most of the nurses are coming from low-income families, they generally value job security as the most important criterion and, therefore, the preference for public sector jobs continues to be high among nurses in this country.

This study also found that factors that significantly influenced students’ preferences in choosing to work in the public sector directly after graduation were: 1) having received high school education in rural areas (OR = 0.79), 2) their parents are living in rural areas (OR = 0.79), and 3) a student’s father having a higher level of education (OR = 1.17). This finding is consistent with a study conducted in Thailand which found that special-track medical graduates, admitted through a rural recruitment program targeting rural high school students, had a 10% higher tendency to prefer working in rural areas compared to those recruited through the conventional, national entrance exam, track [5]. Another study in Thailand also found that the special track medical students had a 10 to 15% higher probability of completing their mandatory service in rural areas [6]. This may also be because public health services in these Asian countries are normally located in rural areas. Therefore, most students intended to opt to work in the public sector in order to be able to live with their parents in a familiar environment.

### Strengths and limitations of the study

The large scale of the survey in each country provided a statistical power for the analysis. Furthermore, it should be noted that all the countries which participated in this study developed a common protocol together. Therefore, variations of the study data due to a country’s specific context would have been minimized as all countries strictly followed the same protocol. In addition, although this study employed a centralized database and pooled data analysis, individual country data would be further explored during the analysis if there were any country specific issues that needed a clearer explanation. The study also faced some limitations. Firstly, because it employed a cross-sectional, self-assessment survey conducted among nursing students, it was not possible to follow-up whether the students’ jobs after graduation were actually the same as predicted by this study’s model. Secondly, social desirability bias may be an issue as some students might provide answers in favor of social expectations. However, our data collectors were well-trained to explain that all responses would be kept strictly confidential. Hence, this bias would have been minimized. Lastly, there may be some other factors affecting students’ attitudes, intention, and competencies that we did not include in this study (e.g. parental guidance, type of training experiences, intention to work in or out of their home countries). Thus, further qualitative research that explores the experience of nursing students during their childhood and training period and how this affects the graduates’ attitude, professional competencies, clinical skills, and intention to work upon graduation might be useful for future curriculum development and HRH planning.

## Conclusion and recommendations

Findings from the five Asian countries studied confirmed that nursing students with rural upbringings and recruitment had more positive attitudes toward rural areas and were more likely to choose to work in rural areas after graduation. This study provides additional evidence from country implementation to support the strength of WHO recommendations on effective strategies to address rural retention, which should have been focusing on rural background as well as recruitment. In addition, nursing curricula should provide more training activities and exposure to rural practice and environments to build up students’ positive attitudes towards working in rural areas, as well as to allow them to gain more confidence in the competencies necessary for working after graduation.

## Abbreviations

ANHER: Asia-pacific network for health professional education reforms
ANM: Auxiliary nurse midwife
BGD: Bangladesh
BHPI: Bangladesh Health Professions Institute
BSc: Bachelor of ccience
CCU: Coronary care unit
CHN: China
CRP: Centre for the rehabilitation of the paralysed
GNM: General nursing and midwifery
HRH: Human resources for health
ICU: Intensive care unit
IND: India
LMICs: Low-middle income countries
MOPH: Ministry of public health
RNs: Registered nurses
THA: Thailand
VNM: Vietnam
WHO: World Health Organization
WHO-SEARO: WHO-Southeast Asia regional office
